# The Main Anticancer Bullets of the Chinese Medicinal Herb, Thunder God Vine

**DOI:** 10.3390/molecules16065283

**Published:** 2011-06-23

**Authors:** Zi Liu, Liang Ma, Guang-Biao Zhou

**Affiliations:** 1 Division of Molecular Carcinogenesis and Targeted Therapy for Cancer, State Key Laboratory of Biomembrane and Membrane Biotechnology, Institute of Zoology, Chinese Academy of Sciences, Beijing 100101, China; 2 School of Life Sciences, University of Science and Technology of China, Hefei 230027, China

**Keywords:** Thunder god vine/*Tripterygium wilfordii* Hook. f., triptolide, celastrol, anticancer activity

## Abstract

The thunder god vine or *Tripterygium wilfordii* Hook. F. is a representative Chinese medicinal herb which has been used widely and successfully for centuries in treating inflammatory diseases. More than 100 components have been isolated from this plant, and most of them have potent therapeutic efficacy for a variety of autoimmune and inflammatory diseases. In the past four decades, the anticancer activities of the extracts from this medicinal herb have attracted intensive attention by researchers worldwide. The diterpenoid epoxide triptolide and the quinone triterpene celastrol are two important bioactive ingredients that show a divergent therapeutic profile and can perturb multiple signal pathways. Both compounds promise to turn traditional medicines into modern drugs. In this review, we will mainly address the anticancer activities and mechanisms of action of these two agents and briefly describe some other antitumor components of the thunder god vine.

## 1. Introduction

**Figure 1:**
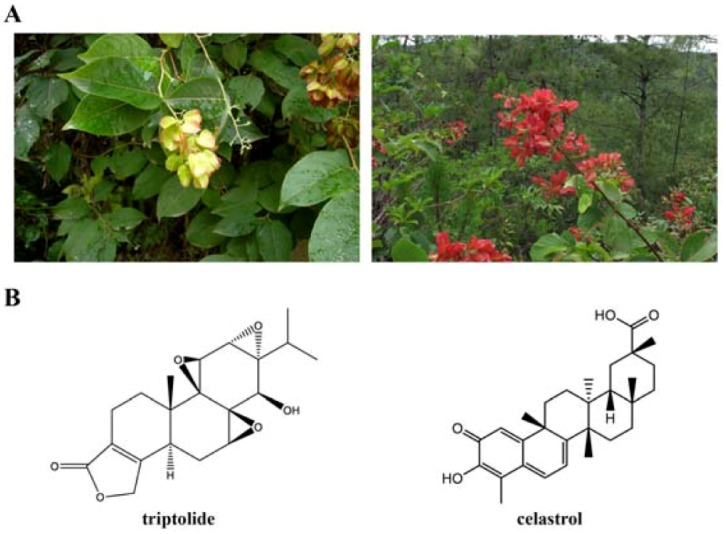
*Tripterygium wilfordii* Hook F, triptolide and celastrol. (**A**): *Tripterygium wilfordii* Hook F. images courtesy of Dr. Yong-Xian Cheng at Kunming Institute of Botany, Chinese Academy of Sciences. (**B**): Chemical structure of triptolide (left) and celastrol (right).

Traditional Chinese Medicine (TCM), which has been used for centuries in treating illnesses ranging from inflammation to cancer, continues to provide front-line pharmacotherapy for many millions of people worldwide. Evidence records that compounds from medicinal herbs and minerals are considered as the source or inspiration for the majority of FDA-approved agents [1,2]. As a representative of Chinese medicinal herb, thunder god vine (*Tripterygium wilfordii* Hook. f., TwHf; also known as Lei Gong Teng, seven-step vine, Figure 1A) which belongs to genus *Tripterygium*, *Celastraceae* family, grows widely in the mountainous regions of southeast and southern China. A large body of knowledge demonstrates the promising therapeutic potentials of TwHf in a number of autoimmune and inflammatory conditions, and phase 2b clinical trials have been conducted to test the efficacy of TwHf extracts in rheumatoid arthritis [3,4], Crohn’s disease [5] and kidney transplantation [6]. Adverse effects such as diarrhea, headache, nausea and infertility are also recorded, and numerous attempts have been made to improve its efficacy and safety [7]. 

A broad spectrum of therapeutic profile of TwHf may be attributed to its complex mixture of ingredients. TwHf contains more than 100 small compounds such as diterpenes, triterpenes, sesquiterpenoids, and alkaloids, which are used for the treatment of a variety of autoimmune and inflammatory diseases including rheumatoid arthritis, nephritis, and systemic lupus erythematosus [8,9]. Some components of TwHf exhibit powerful anti-fertility effects in male animal models [10,11]. Recently, researchers worldwide have paid more attention on the anticancer activities of TwHf extracts, with triptolide and celastrol as the two most promising and potent bioactive ingredients [12,13]. Though advances have been made in understanding the molecular mechanisms of action of these two compounds, the exact targets of triptolide and celastrol remain elusive, and they must pass along a pathway of chemical synthesis, mechanistic studies and clinical testing before their eventual deployment in the clinic.

## 2. Triptolide

Triptolide (Figure 1B) is a diterpenoid triepoxide firstly purified from the roots of TwHf in 1972 [14], and several synthetic routes have been described [15,16]. Reports document that triptolide has anti-inflammatory and immunosuppressive, anti-fertility and anticancer abilities [14]. Triptolide has been tested in clinical trials for the treatment of psoriasis vulgaris [17], diabetic nephropathy [18] and nephritic syndrome [19]. On the other hand, many derivatives of triptolide have been synthesized to improve water solubility and reduce the potential side effects. For example, PG490-88 [20] is a succinate salt water-soluble derivative which has entered Phase I clinical trials as an immunosuppressant.

### 2.1. Antitumor Activity of Triptolide

It is well documented that triptolide has a broad spectrum ability to inhibit proliferation and induce apoptosis of various cancer cell lines *in vitro* and prevent tumor growth and metastases *in vivo*. Triptolide shows anticancer activity in cells derived from both hematological malignancies and solid tumors, such as HL-60, T cell lymphoma (Jurkat) [21], U937, OCI-AML3 [22], Kasumi-1 and SKNO-1 cells [23], human hepatocelluar carcinoma SMMC-7721 cells, and cell lines of multiple myeloma, breast, gastric, prostate, lung, oral, colon, pancreatic and cervical cancers [24,25,26,27,28], cholangiocarcinoma [29], and neuroblastoma [30,31]. The *in vivo* experiments have also demonstrated triptolide’s therapeutic efficacy in several model systems including cholangiocarcinoma in a hamster model [29] and xenografts of human melanoma, breast cancer, bladder cancer, gastric carcinoma [32], pancreatic cancer [33] and neuroblastoma in nude mice [34]. 

### 2.2. Mechanisms of Action

**Figure 2:**
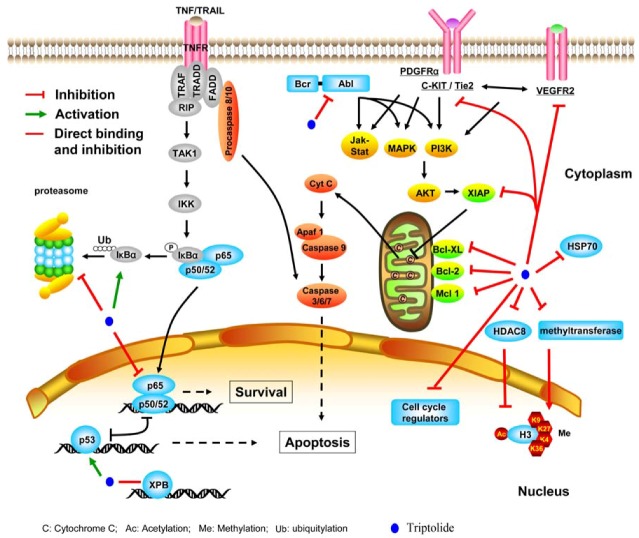
Schematic represents the main molecular mechanisms of action of triptolide.

Molecular mechanisms underlying triptolide’s anticancer activity have been extensively investigated, and reports show that triptolide is capable of interfering with a variety of signal pathways, many of which are crucial for the survival of cancer cells (Figure 2).

#### 2.2.1. Targeting transcription factors and epigenetic modifiers

Since triptolide has epoxide moieties, it is conceivable that this compound could bind to a certain cellular protein via formation of covalent bond. In 1974, Kupchan *et al*. [35] suggested that the 14b hydroxyl along with the 9,11 epoxide might be responsible for the observed antitumor activity. In 2007, McCallum *et al*. [36] discovered that triptolide could bind specifically and irreversibly through the epoxide moieties to a 90 kDa nuclear protein, which may be a transcriptional regulator or somehow involved in turnover of a critical transcriptional regulator, such that its covalent modification prevented a key step in transcription. Recently, Titov *et al*. [37] reported that triptolide covalently bound to a human 90 kD protein, XPB (also known as ERCC3) which is a subunit of the transcription factor TFIIH, and inhibited its DNA-dependent ATPase activity, which led to the inhibition of RNA polymerase II–mediated transcription and likely nucleotide excision repair. The identification of XPB as the target of triptolide accounts for the majority of the known biological activities of triptolide.

In human gastric and prostatic epithelial cells [24] and HL-60 leukemia cells [38], triptolide-caused proliferation inhibition and apoptosis induction may be primarily mediated by its modulation of p53, a nuclear phosphoprotein which acts as a tumor suppressor. Nuclear factor κB (NF-κB) is a transcription factor that can promote cell survival, stimulate growth, and reduce susceptibility to apoptosis via upregulation of various targeted proteins. Inhibition of NF-κB may lead to cell apoptosis [39]. Lee *et al.* [40] showed that triptolide blocked TNF-induced NF-κB activation via inhibiting p65 transactivation but not DNA binding affinity, thus promoting TNF-triggered apoptosis. However, other reports suggest that triptolide inhibits the DNA binding ability of NF-κB or cytokine-stimulated NF-κB activity [40,41,42]. In multiple myeloma cells, triptolide decreases histone H3K9 and H3K27 methylation via the downregulation of histone methyltransferase SUV39H1 and EZH2, respectively, and reduces the expression of HDAC8, leading to increase of the histone H3 and H4 acetylation [43]. Triptolide also inhibits the activity of RNA polymerase, resulting in the general transcription inhibition [44].

#### 2.2.2. Inhibiting molecular chaperone and proteasome

Molecular chaperone heat shock protein (HSP) represents a group of highly conserved proteins that can protect cells from adverse environmental, physical and chemical stresses, and is reported as inhibitors of apoptosis [45]. By a small molecule screening assay, Westerheide *et al.* [46] recently demonstrated that triptolide was an inhibitor of human heat shock gene transcription which led to enhancement of stress-induced cell death. As a major stress-inducible HSP, HSP70 renders cells highly resistant to several chemotherapeutic drugs. Interestingly, Philips *et al*. [33] reported that triptolide could inhibit HSP70 at both mRNA and protein levels, and induce apoptosis in pancreatic cancer cells with overexpressed HSP70. 

The ubiquitin/proteasome system is an important cellular pathway for protein degradation. Given that the aromatic ketone carbon could interact with the hydroxyl group at the N-terminal threonine of the β5 subunit of the proteasome, thus causing inhibition of the proteasomal chymotrypsin-like activity [13,47], triptolide which forms ketones under oxidizing conditions might have potential proteasome inhibitory activity. This possibility was confirmed by a recent study [48] which showed that triptolide could inhibit cellular proteasomal chymotrypsin-like activity, resulting in accumulation of proteasomal substrates including IκB, p27 and Bax, and subsequently apoptosis of both PC-3 and MDA-MB-231 cancer cells.

#### 2.2.3. Suppressing kinases

Gain-of-function mutation of C-KIT, a member of the type III receptor tyrosine kinase family, activates its downstream pathways (Jak-STAT, MAPK, and PI3K) and confers uncontrolled cell proliferation and survival advantages to cancer cells [49,50]. C-KIT abnormalities are closely associated with acute myeloid leukemia (AML) with t(8;21) [49], the most common chromosomal translocation seen in AML which generates the AML1-ETO (RUNX1-RUNXT1) fusion transcript. AML1-ETO may upregulate C-KIT via inactivation of TGFβ [49]. Recently, Zhou *et al*. [23] showed that triptolide triggered inactivation of C-KIT and a caspase-3-dependent cleavage of AML-ETO, forming a positive feedback loop to induce programmed cell death of t(8;21) leukemic cells. Jin *et al*. [51] reported that triptolide inhibited imatinib-resistant mast cells harboring D816V C-KIT. Suppression of other kinases as Bcr-Abl, PDGFRα, and Jak2 by triptolide was also related to its anti-cancer activity [52,53,54]. In addition, triptolide was shown to be able to inhibit tumor angiogenesis through regulating VEGFR-2 and Tie2 angiogenic pathways [55].

#### 2.2.4. Perturbing other molecules

In a cDNA array analysis, Zhao *et al*. [56] demonstrated that triptolide inhibited the expression of genes involved in cell cycle progression and cell survival, such as cyclins D1, B1, and A1, Cdc-25; Bcl-X and c-Jun. Triptolide reduced the expression of apoptosis antagonists XIAP, Bcl-2 and Mcl-1 [20]. Triptolide induced caspase-dependent apoptosis of leukemia and cervical cancer cells [28,57], and triggered caspase-independent autophagic cell death in pancreatic cancer cells [30]. Leuenroth *et al*. [58] identified calcium (Ca^2+^) channel polycystin-2 (PC2) as a putative direct target of triptolide in a mouse model of polycystic kidney disease (PKD). Triptolide may perturb multiple targets and interfere with multiple signaling pathways, and potentiate activities of other antitumor agents such as Apo2/TRAIL, tumor necrosis factor α, and other chemotherapeutic agents. 

## 3. Celastrol

### 3.1. The Antitumor Activity

Celastrol or tripterine (Figure 1B) is rich in root skin and bark of TwHf and was the first monomer extracted from the root of TwHf in 1936 [59,60,61]. As a triterpenoid, celastrol is structurally differed from other TwHf extracts. Celastrol exhibits potent anti-inflammatory activities in various experimental animal models, including collagen-induced arthritis [62], amyotrophic lateral sclerosis [63], Alzheimer’s disease [64,65], lupus [62,66,67], asthma [68,69], and rheumatoid arthritis [62,70]. Celastrol also has potent antitumor activities. It inhibits proliferation and induces apoptosis in various cancer cell lines, such as those derived from leukemia [71,72,73,74,75,76], pancreatic [77,78], gliomas [79,80,81,82], prostate [13,83,84,85] and breast [86,87,88] cancers, and has the ability to suppress invasion and angiogenesis of tumor cells [81,82]. Celastrol sensitizes melanoma cells to temozolomide treatment [89], facilitates mitotic cell death triggered by microtubule-targeting anti-cancer drugs [90], potentiates TNF [91] or TRAIL [92] -induced apoptosis, and enhances the effects of radiotherapy in prostate [83] and lung cancer cells [93]. 

### 3.2. Mechanisms of Action

#### 3.2.1. Celastrol interferes with multiple signal pathways

**Figure 3:**
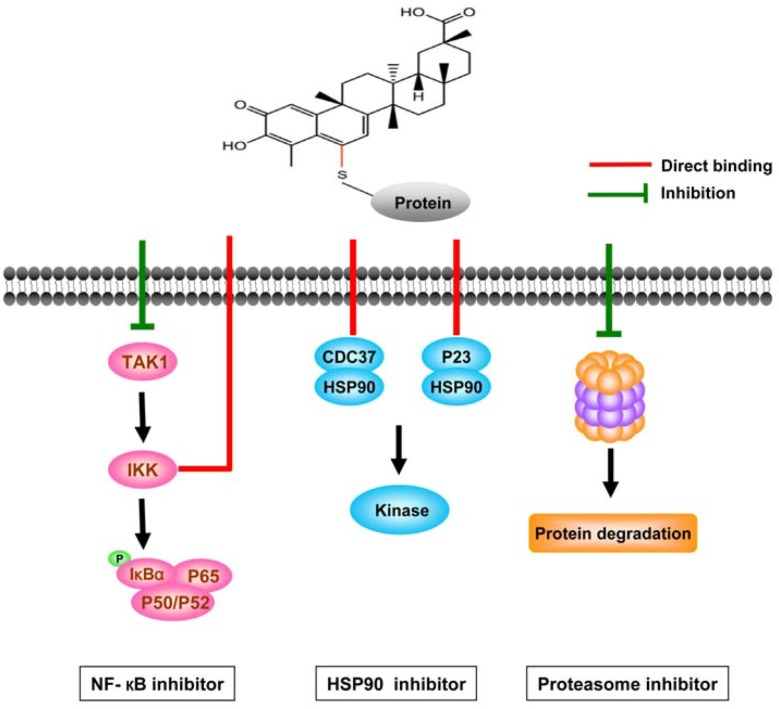
Celastrol targets three major signal pathways for its antitumor effects.

Plenty of work has been done focusing on mechanisms of action of celastrol, and the major pathways affected are shown in Figure 3. Celastrol inhibits NF-κB through targeting IκB kinase and TAK1-induced NF-κB activation [91,94], binds to Cdc37 and disrupts the Cdc37–Hsp90N complex which is critical for stabilizing oncogenic kinases in various cancers [78,95,96], and inactivates the p23 protein which is another co-chaperone of HSP90 [97]. Celastrol also inhibits topoisomerase II [98], potassium channels [99], and AKT/Mammalian target of rapamycin pathway [85]. It suppresses cell-extracellular matrix adhesion via targeting β1 integrin [100], down-regulates the expression of VEGF receptor [82] and cell survival proteins and up-regulates death receptors via the ROS-mediated increase of CHOP pathway [92]. 

Celastrol was shown to be able to inhibit the proteasome activity and accumulate the ubiquitinated proteins in prostate cancer cell lines [13,101]. However, Chapelsky *et al*. [102] reported that in RAW264.7 cells, celastrol showed slight inhibitory activity against the chymotryptic activity of the 20S proteasome at high (10 µM) but not low (3 µM) concentration, and did not inhibit the chymotrypsin-like activity of the 26S proteasome which is responsible for the degradation of ubiquitylated proteins in intact cells. Celastrol cannot inhibit the cleavage of the substrates by the 26S proteasome, making it very different from other proteasome inhibitors such as MG-132 [102]. Celastrol-caused accumulation of ubiquitinated proteins may be a result of HSP90 inhibition and stress response. In contrast to other proteasome inhibitors, celastrol-induced inhibition of IκB-α degradation is due to its suppression of IκB-α phosphorylation [91]. 

#### 3.2.2. Direct targets of celastrol

Since celastrol possesses a broad range of biological activity, it is crucial to identify its direct targets. Structure-activity studies indicate that the quinone methide functional group of celastrol may be responsible for its cytotoxic activity [13,103]. Computational electron density analysis demonstrates that C2 on A-ring and C6 on B-ring of celastrol have a high susceptibility toward a nucleophilic attack, suggesting that one or both of these carbons could interact with its target proteins [78,96,103]. Indeed, studies show that celastrol can interact with the nucleophilic thiol groups of cysteine residues and form covalent Michael adducts. The two primary functions of celastrol, inhibition of the HSP90 and suppression of NF-kB pathway, may be attributed to its ability to interact with the thiol groups of cysteine residues of the proteins. The inhibition of NF-kB activation by celastrol could be abolished by dithiothreitol (DTT) and reduction of the quinone methide of celastrol with NaBH_4_ to dihydrocelastrol [94]. When all the nine cysteine residues of full-length Cdc37 are blocked with N-ethylmaleimide (NEM), it no longer reacts with celastrol, indicating that cysteines indeed undergo chemical reactions with celastrol [96]. Additionally, it was also reported that the effects of celastrol could be countered by pre-loading thiol-containing agents and celastrol and thiol-containing agents could also react with each other to form new compounds [74]. Another study further confirms that the quinone methide moiety seems crucial to celastrol’s effects on melanoma cells because dihydrocelastrol which lacks this moiety, fails to inhibit melanoma cell viability, whereas pristimerin, the celastrol methyl ester with the quinone methide functional group retained intact in the molecules, is equipotent or even slightly more potent than celastrol against SW1 cells. These findings strongly suggest that celastrol could bind to proteins irreversibly or pseudo-irreversibly in melanoma cells, possibly through interaction with cysteinyl residues as well [104]. Taken together, these results indicate that celastrol may affect the functions of a variety of proteins via formation of Michael adducts, which seems to be the major mechanism contributing to its broad anticancer effects.

### 3.3. Prospective

Despite these advances, some questions still need to be further addressed. Firstly, as a HSP90 inhibitor, celastrol decreases many HSP90 client proteins including Akt, Cdk4, FLT3, EGFR, BCR-ABL and androgen receptor (AR), but the mechanisms underlying remain largely unknown [78,95]. Secondly, it was showed that celastrol could inhibit p23 function by altering its 3-dimensional structure, leading to rapid formation of amyloid-like fibrils. This may be triggered by the non-covalent binding of celastrol to p23, rather than irreversibly reacting with cysteine residues in p23, though covalent interaction indeed forms between them [97]. However, evidence is needed to elucidate this possibility. Moreover, routes for chemical synthesis of celastrol are desired to reduce reliance on the natural source which are critical for drug development [1,103]. Finally, celastrol is one of the main ingredients with reproductive toxicity, which may greatly limit its application. Thus, new derivatives and analogues of celastrol with higher pharmacological activities and lower toxicological effects should be designed and synthesized.

## 4. Other Antitumor Components

In addition to triptolide and celastrol, other extracts possessing anticancer activity have also been isolated from TwHf. Tripdiolide, an alcohol extract of TwHf, was reported to exhibit antileukemia activity in 1972 [14]. Five diterpenes from TwHf, 3-epi-triptobenzene B (I), 3β,14- dihydroxyabieta-8,11,13-triene (triptobenzene B, II), wilforol E (III), triptohairic acid (IV) and 11-hydroxy-14-methoxy-18(4→3)-abeo-abietan-3,8,11,13-tetraen-18-oic-acid (hypoglic acid, V), present antitumor activity on Hela and L292 cell lines [105]. Three triterpene components, triptotin G, wilforol A, and triptotin D, also show strong inhibitory activity against leukemia and lung cancer cell lines [106].

## 5. Conclusion Remarks

As prospective anti-tumor drug candidates, TwHf and its extracts have been studied widely in the past several decades. However, there are also many challenges warranting careful investigation. It is widely accepted that the main active components of TwHf are triptolide and celastrol. However, due to the complexity of the chemical ingredients of TwHf, it will be valuable to detect if there are any other more effective active components for cancer therapy. In addition, the precise molecular mechanisms of triptolide and celastrol remain obscure, which may also be important to elucidate the potential mechanisms of toxicity and to design and synthesize compounds or derivatives with more selective activity and to reduce the unexpected therapeutic side effects. Finally, it is vital to explore the absorption, distribution, metabolism, excretion, and toxicity (ADMET) characteristics of these monomer compounds extracted from TwHf, laying a sound foundation for clinical trails in the disease interested in the future.
